# A Non Mouse-Adapted Dengue Virus Strain as a New Model of Severe Dengue Infection in AG129 Mice

**DOI:** 10.1371/journal.pntd.0000672

**Published:** 2010-04-27

**Authors:** Grace K. Tan, Jowin K. W. Ng, Scott L. Trasti, Wouter Schul, George Yip, Sylvie Alonso

**Affiliations:** 1 Department of Microbiology, Immunology Programme, National University of Singapore, Singapore, Singapore; 2 Comparative Medicine Centre, National University of Singapore, Singapore, Singapore; 3 Novartis Institute for Tropical Diseases (NITD), Singapore, Singapore; 4 Department of Anatomy, National University of Singapore, Singapore, Singapore; University of California, Berkeley, United States of America

## Abstract

The spread of dengue (DEN) worldwide combined with an increased severity of the DEN-associated clinical outcomes have made this mosquito-borne virus of great global public health importance. Progress in understanding DEN pathogenesis and in developing effective treatments has been hampered by the lack of a suitable small animal model. Most of the DEN clinical isolates and cell culture-passaged DEN virus strains reported so far require either host adaptation, inoculation with a high dose and/or intravenous administration to elicit a virulent phenotype in mice which results, at best, in a productive infection with no, few, or irrelevant disease manifestations, and with mice dying within few days at the peak of viremia. Here we describe a non-mouse-adapted DEN2 virus strain (D2Y98P) that is highly infectious in AG129 mice (lacking interferon-α/β and -γ receptors) upon intraperitoneal administration. Infection with a high dose of D2Y98P induced cytokine storm, massive organ damage, and severe vascular leakage, leading to haemorrhage and rapid death of the animals at the peak of viremia. In contrast, very interestingly and uniquely, infection with a low dose of D2Y98P led to asymptomatic viral dissemination and replication in relevant organs, followed by non-paralytic death of the animals few days after virus clearance, similar to the disease kinetic in humans. Spleen damage, liver dysfunction and increased vascular permeability, but no haemorrhage, were observed in moribund animals, suggesting intact vascular integrity, a cardinal feature in DEN shock syndrome. Infection with D2Y98P thus offers the opportunity to further decipher some of the aspects of dengue pathogenesis and provides a new platform for drug and vaccine testing.

## Author Summary

The spread of dengue (DEN) worldwide combined with an increased severity of the DEN-associated clinical outcomes have made this mosquito-borne virus of great global public health importance. Infection with DEN virus can be asymptomatic or trigger a wide spectrum of clinical manifestations, ranging from mild acute febrile illness to classical dengue fever and to severe DEN hemorrhagic fever/DEN shock syndrome (DHF/DSS). Progress in understanding DEN disease and in developing effective treatments has been hampered by the lack of a suitable animal model that can reproduce all or part of the disease's clinical manifestations and outcome. Only a few of the DEN virus strains reported so far elicit a virulent phenotype in mice, which results at best in an acute infection where mice die within few days with no, few or irrelevant disease manifestations. Here we describe a DEN virus strain which is highly virulent in mice and reproduces some of the aspects of severe DEN in humans, including the disease kinetics, organ damage/dysfunction and increased vascular permeability. This DEN virus strain thus offers the opportunity to further decipher some of the mechanisms involved in DEN pathogenesis, and provides a new platform for drug and vaccine testing in the mouse model.

## Introduction

Dengue (DEN) virus belongs to the *Flaviviridae* family, *Flavivirus* genus, and is the causative agent of DEN disease, a mosquito-borne illness that is endemic in subtropical and tropical countries [1]. With approximately half of the world's population residing in DEN endemic regions [2] and more than 50 million new infections projected to occur annually [3], DEN certainly poses as a global economic and health threat.

Infection with one of the four DEN serotypes can be asymptomatic or trigger a wide spectrum of clinical manifestations, ranging from mild acute febrile illness to classical dengue fever (DF), and to severe dengue hemorrhagic fever/dengue shock syndrome (DHF/DSS), characterized by fever, hemorrhagic tendency, thrombocytopenia, and capillary leakage according to the WHO guidelines [4]. Despite the increasing attention and research efforts devoted to DEN in recent years, the cellular and molecular mechanisms responsible for DEN pathogenesis remain largely unknown. Current hypotheses for the development of severe DEN that involve dysfunction of the host immune system include enhancing mechanisms induced by sub-neutralizing cross-reactive antibodies and memory T cells [3], [5]. Other non-enhancing mechanisms implicating the immune system include auto-immune responses against cross-reactive viral components, such as DEN non-structural 1 (NS1) protein [6], [7]. Platelet lysis, nitric oxide-mediated apoptosis of endothelial cells and complement activation have also been proposed to mediate thrombocytopenia and vascular leakage [8]. In addition, host genetic predisposition [9]–[11] and virus virulence [12], [13] were reported as risk factors for the development of severe DEN.

No effective drugs or vaccines against DEN are currently available on the market [14]. Undeniably, progress in deciphering the mechanisms responsible for DEN pathogenesis and in developing effective prophylactic and/or therapeutic treatments has been impeded by the lack of suitable animal models [15]. Humans and mosquitoes represent so far the only natural hosts for DEN virus. Non-human primates have been reported to be permissive to DEN infection but no apparent clinical symptoms of the disease were observed [16], [17], although a recent study reported signs of hemorrhage in rhesus macaques intravenously infected with a high dose of a DEN2 virus strain [18]. In addition, since the infected animals develop transient viremia and antibody responses, they have been useful for evaluating the efficacy of vaccine and antiviral candidates prior to clinical trials in humans [19], [20]. However, for ethical and economical reasons, non-human primates do not represent a sustainable option for DEN research. Alternatively, the mouse model has been explored [15]. However, most of the DEN virus laboratory strains and clinical isolates do not replicate efficiently in mice. Mouse-adapted DEN virus strains displayed a higher infectivity but led to irrelevant clinical manifestations such as paralysis [21], [22]. Alternatively, a variety of mouse genetic backgrounds have been explored that displayed greater susceptibility to DEN infection [23]–[30]. Among them, AG129 mice, deficient in interferon (IFN)- α/β and -γ receptors, were shown to allow effective replication of DEN virus [30]–[33]. However, great heterogeneity in the susceptibility of these mice to DEN virus strains, even within the same serotype, was reported [32] with none or few of DEN disease manifestations [30]. Moreover, administration of high viral doses was necessary to trigger a virulent phenotype which resulted in the animals' death within few days at the peak of viremia [30]. This is in contrast to humans for whom signs of severe DEN generally occur during or after defervescence when DEN virus is no longer detectable in the patient's blood [3], [34], [35].

Here we describe a unique non mouse-adapted strain of DEN virus serotype 2 (D2Y98P) which is highly infectious in AG129 mice upon intraperitoneal administration. Infection with a high viral dose of D2Y98P resulted in an acute model of infection with mice dying at the peak of viremia, whereas infection with a low viral dose led to asymptomatic dissemination and replication of the virus followed by death of the animals after the virus has been cleared from its host.

## Materials and Methods

### Ethics statement

All the animal experiments were carried out under the guidelines of the National University of Singapore animal study board.

### Virus strain and growth conditions

The virus strain used in this study (D2Y98P) derives from a 1998 DEN2 Singapore human isolate that has been exclusively passaged for about 20 rounds in *Aedes albopictus* C6/36 cells. C6/36 cells (ATCC# CRL-1660) were maintained in Leibovitz's L-15 medium (GIBCO) supplemented with 5% fetal calf serum (FCS), and virus propagation was carried out as described previously [32]. Virus stocks were stored −80°C. When necessary, heat-inactivation of the virus was performed at 55°C for 15 min.

### Virus quantitation

Plaque assay was carried out to quantify the number of infectious viral particles using BHK-21 (Baby Hamster Kidney, ATCC# CCL-10) cells as described previously [36] with slight modifications. Briefly, BHK cells were cultured to approx. 80% confluency in 24-well plates (NUNC, NY, USA). The virus stock was 10-fold serially diluted from 10^−1^ to 10^−8^ in RPMI 1640 (GIBCO). BHK-21 monolayers were infected with 100 ul of each virus dilution. After incubation at 37°C and 5% C0_2_ atmosphere for 1 hr with rocking at 15 min intervals, the medium was decanted and 1 ml of 1% (w/v) carboxymethyl cellulose in RPMI supplemented with 2% FCS was added to each well. After 4 days incubation at 37°C in 5% CO_2_, the cells were fixed with 4% paraformaldehyde and stained for 30 min with 200 µl of 1% crystal violet dissolved in 37% formaldehyde. After thorough rinsing with water, the plates were dried and the plaques were scored visually.

### Mice infection

AG129 [129/Sv mice deficient in both alpha/beta (IFN-α/β) and gamma (IFN-γ) interferon receptors] were obtained from B&K Universal (UK). They were housed under specific pathogen-free conditions in individual ventilated cages. Eight to 9 week-old mice were administered with 10^7^ to 10^2^ plaque forming units (PFU) of D2Y98P via the intraperitoneal (ip.) route (0.4 ml in sterile PBS). Where indicated, mice were inoculated with the same dose and volume of heat-inactivated D2Y98P.

### Antibody titres

Systemic antibody titres against D2Y98P were determined by enzyme-linked immunoadsorbent assay (ELISA) as described previously [32]. Briefly, 96-well plates (Corning costar, NY, USA) were coated overnight at 4°C with 10^5^ PFU of heat-inactivated D2Y98P virus in 0.1M NaHCO3 buffer at pH 9.6. Two-fold serially diluted serum samples (1∶25 to 1∶25,600) were added to the wells and incubated for 1 hr at 37°C. HRP-conjugated anti-mouse IgM (Chemicon) or IgG (H+L) (Bio-rad) secondary antibody were used at a 1∶3,000 dilution. Detection was performed using Sigma*Fast*™ O-phenylenediamine dihydrochloride substrate (Sigma Aldrich) according to the manufacturer's instructions. The reaction was stopped with 75 µl of 1M H_2_SO_4_ and absorbance was read at 490 nm using an ELISA plate reader (Bio-rad model 680). ELISA titres were defined as the reciprocal of the highest serum dilution that equals to 3 times the absorbance reading from uninfected mouse serum sample.

### Plaque reduction neutralization test (PRNT)

PRNT was carried out as described previously [36] with modifications. Briefly, mouse serum samples were heated at 56°C for 30 min to inactivate complement. Two-fold serial dilutions of the sera (1∶10 to 1∶10,240 in RPMI 1640) were mixed in 96-well plates with an equal volume containing 30 PFU of D2Y98P, and incubated at 37°C for 1 hr with rocking every 15 min. Each mix (100 µl) was transferred onto BHK monolayers grown in 24-well plates, and incubated at 37°C for 1 hr. The mix was decanted, and plaque assay was carried out as described above. The percentage of plaque reduction was derived relative to the control consisting of virus mixed with uninfected serum: [1- (number of plaques in test wells/number of plaques in control wells) *100]. Fifty percent neutralization titres (PRNT_50_) were determined for each sample by fitting a variable sigmoidal curve in GraphPad Prism 5.00 (GraphPad Software). Data are expressed as the reciprocal of the highest serum dilution for which PRNT_50_ is obtained.

### Determination of virus titres in infected mice

Blood samples were collected in 0.4% sodium citrate and centrifuged for 5 min at 6,000 *g* to obtain plasma. The presence of infectious viral particles was determined by plaque assay as described above.

To assess the levels of infectious virus in the tissues from infected mice, the animals were euthanized and perfused systemically with 50 ml sterile PBS. Whole tissue from the brain, intestines, liver and spleen were harvested from individual mice, kept on ice and their wet weights were recorded prior to any further processing. Samples were then trimmed and homogenized using a mechanical homogenizer (Omni) for 5 minutes in 1 ml RPMI 1640 at medium speed on ice. Thoroughly homogenized tissues were clarified by centrifugation at 14,000 rpm for 10 min at 4°C to pellet debris. The supernatant was filter-sterilized using a 0.22 µm diameter pore size filter and the volume was recorded. The level of infectious virus within the filtrate is thus considered representative of the total level of infectious virus present in the harvested organ. Ten-fold serial dilutions of each filtrate (from neat to 1∶10^5^) were assayed in a standard virus plaque assay on BHK-21 cells as described above. Triplicate wells were run for each dilution of each sample. Data are finally expressed as log_10_ [mean ± SD] in PFU per gram of wet tissue with a limit of sensitivity set at 1.0 log_10_ PFU/g of tissue. Five mice per time point per group were assessed. Results are representative of two experiments.

### Histology

Mice were euthanized, and tissues were harvested and immediately fixed in 10% formalin in PBS. Fixed tissues were paraffin embedded, sectioned and stained with Hematoxylin and Eosin (H&E).

### Vascular leakage assessment

Vascular leakage was assessed using Evans Blue dye as a marker for albumin extravasation as described previously [30], [37] with modifications. Briefly, 0.2 ml of Evans blue dye (0.5% w/v in PBS) (Sigma Aldrich) were injected intravenously into the mice. After 2 hrs, the animals were euthanized and extensively perfused with sterile PBS. Vascular permeability in the tissues was determined visually and quantitatively; the tissues were harvested and weighed prior to dye extraction using N,N-dimethylformamide (Sigma; 4 ml/g of tissue wet weight) at 37°C for 24 hrs after which absorbance was read at 620 nm. Data are expressed as fold increase in OD_620nm_ per g of tissue wet weight compared to the uninfected control.

### Cytokine detection

Cytokine (IFN-γ, TNF-α and IL-6) expression levels were measured in individual serum samples using individual detection kits (R&D), according to the manufacturer' instructions. After incubation with detection antibodies and streptavidin-PE complexes, absorbance was read at 450 nm. Five mice per group and per time point were used.

### Hematology

Mouse blood samples were collected in K2EDTA and serum tubes (Biomed Diagnostics). Whole blood was immediately analysed for cell counts using automated hematology analyzer Cell Dyn – 3700 (Abbott). Serum alanine (ALT) and aspartate (AST) aminotransferases, and albumin levels were quantified using chemistry analyzer COBAS C111 (ROCHE).

### Statistical analysis

The results were analyzed using the unpaired Student *t* test. Differences were considered significant (*) at *p* value <0.05.

## Results

### Survival rate of D2Y98P-infected AG129 mice

**Figure 1:**
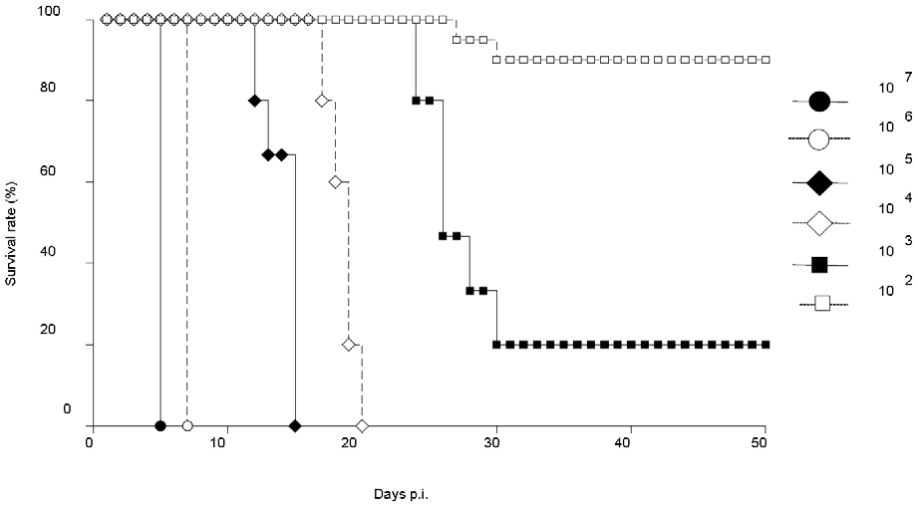
Survival rates in AG129 mice infected with a dose range of D2Y98P virus. AG129 mice were infected intraperitoneally (ip.) with 10-fold serially diluted viral doses of D2Y98P ranging from 10^7^ to 10^2^ PFU. Ten mice per group were used. Data are representative of at least 3 independent experiments.

To test the infectious potential of the D2Y98P strain, AG129 mice were intraperitoneally (ip.) infected with 10-fold serially diluted viral doses ranging from 10^7^ to 10^2^ PFU. Survival rates indicated that infection with 10^4^ PFU and above induced 100% mortality whereas 20% and 90% survival rates were observed in animals infected with 10^3^ and 10^2^ PFU, respectively (Fig. 1). Moreover, in mice infected with lethal doses, a clear correlation between viral dose and time-of-death was observed, with increased heterogeneity as the infectious dose is lower.

**Figure 2:**
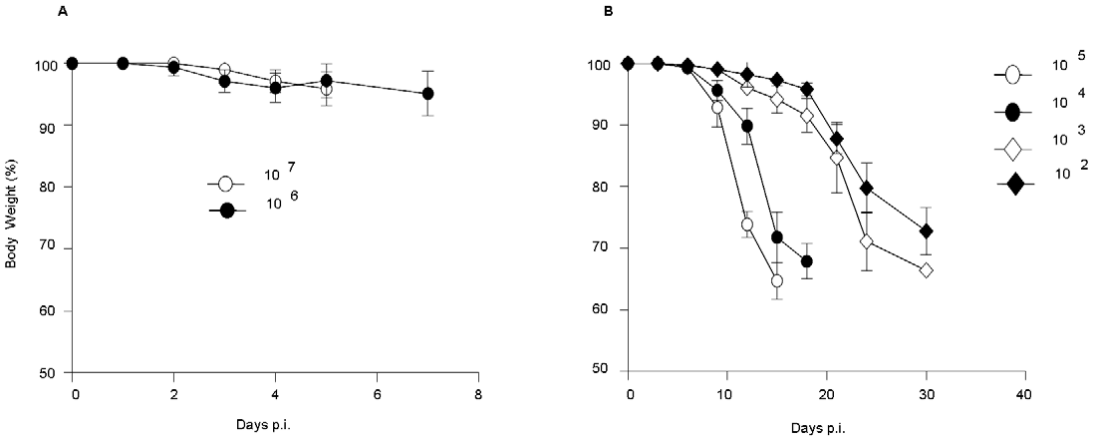
Body weight changes in D2Y98P-infected mice. Mice were ip. infected with 10^7^ or 10^6^ (A), or with 10^5^ to 10^2^ (B) PFU of D2Y98P. Body weight changes were monitored daily (A) or every other day (B) post-infection (p.i.). Results are expressed as the [mean ± SD] of body weight loss in percentage compared to initial body weight. Ten mice per group were monitored. Results are representative of 2 independent experiments.

Upon infection with 10^7^ and 10^6^ PFU, initial clinical signs included ruffled fur and hunched posture, which further progressed to bloatedness, lethargy, diarrhoea-like symptoms, moribund state and finally death of the animals. None of the mice exhibited paralysis or significant body weight loss during the course of infection (Fig. 2A). In contrast, upon infection with 10^5^ PFU and below, no signs of diarrhoea were observed and near moribund state, rapid body weight loss was measured (Fig. 2B).

Mice ip. inoculated with heat-inactivated D2Y98P (10^7^ PFU equivalent) displayed none of the disease manifestations or death. In addition, neither disease manifestation nor transient viremia was observed in immunocompetent Balb/c and C57Bl/6 mice ip. infected with 10^7^ PFU of D2Y98P (data not shown).

### Viremia and antibody titres

Although both viral doses eventually induced 100% mortality in AG129 mice, ip. infection with 10^7^ and 10^4^ PFU of D2Y98P gave very different disease kinetics, suggesting that different mechanisms and players are involved in the disease progression. We thus decided to further characterize both the “acute” and “delayed” models of DEN infection.

**Figure 3:**
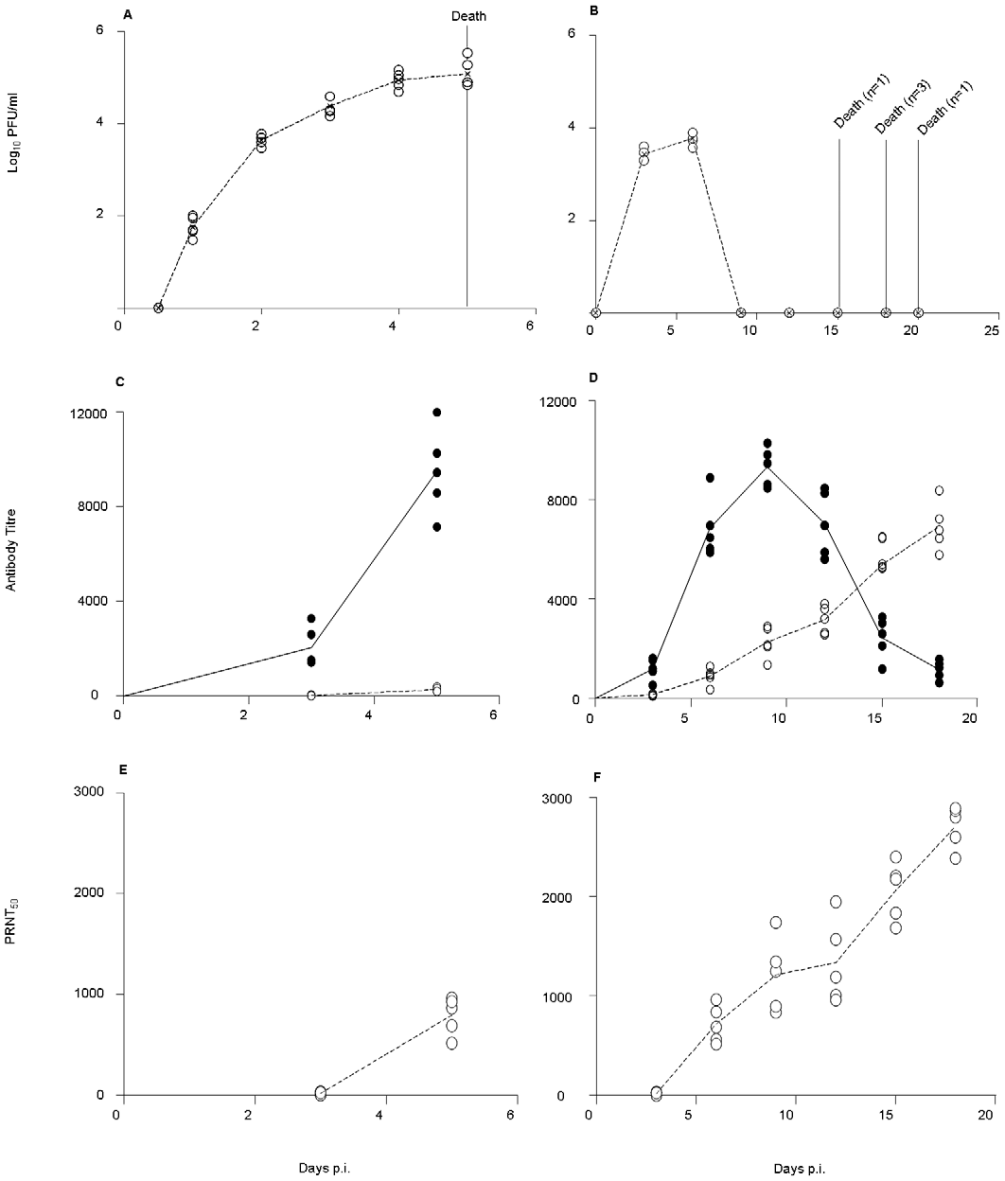
Viremia and antibody titres in D2Y98P-infected mice. Mice were ip. infected with 10^7^ (A, C, E) or 10^4^ (B, D, F) PFU of D2Y98P. At the indicated time points, five infected animals were bled and euthanized immediately. Viremia titres (A, B), specific anti-IgM (black circle) and IgG (open circle) titers (C, D), and PRNT_50_ (E, F) were determined for each individual serum. Results are representative of 2 independent experiments.

Systemic virus titres were monitored over the course of infection for both viral doses. In mice infected with 10^7^ PFU, the peak of viremia (10^5^ PFU/ml) coincided with the animals' death at 5 days p.i. (Fig. 3A). In contrast, in mice infected with 10^4^ PFU, viremia peaked at around 10^4^ PFU/ml at 6 days p.i., followed by viral clearance from the blood circulation prior to animal death (Fig. 3B), similar to the disease kinetic described in severe DEN patients [3], [34], [35], [38].

Furthermore, specific IgM and IgG antibody titres were monitored over the course of infection. Significant IgM but weak IgG responses were measured in mice infected with 10^7^ PFU which both peaked at the time of death, 5 days p.i. (Fig. 3C). Instead, in mice infected with 10^4^ PFU, significant IgG antibody titers were detected which progressively increased over time, while the IgM antibody response peaked at day 10 p.i. and waned by day 18 p.i. (Fig. 3D). Neutralizing antibody titres correlated with the IgG antibody responses (Fig. 3E&F).

### Tissue tropism and kinetic of virus replication in D2Y98P-infected mice

**Figure 4:**
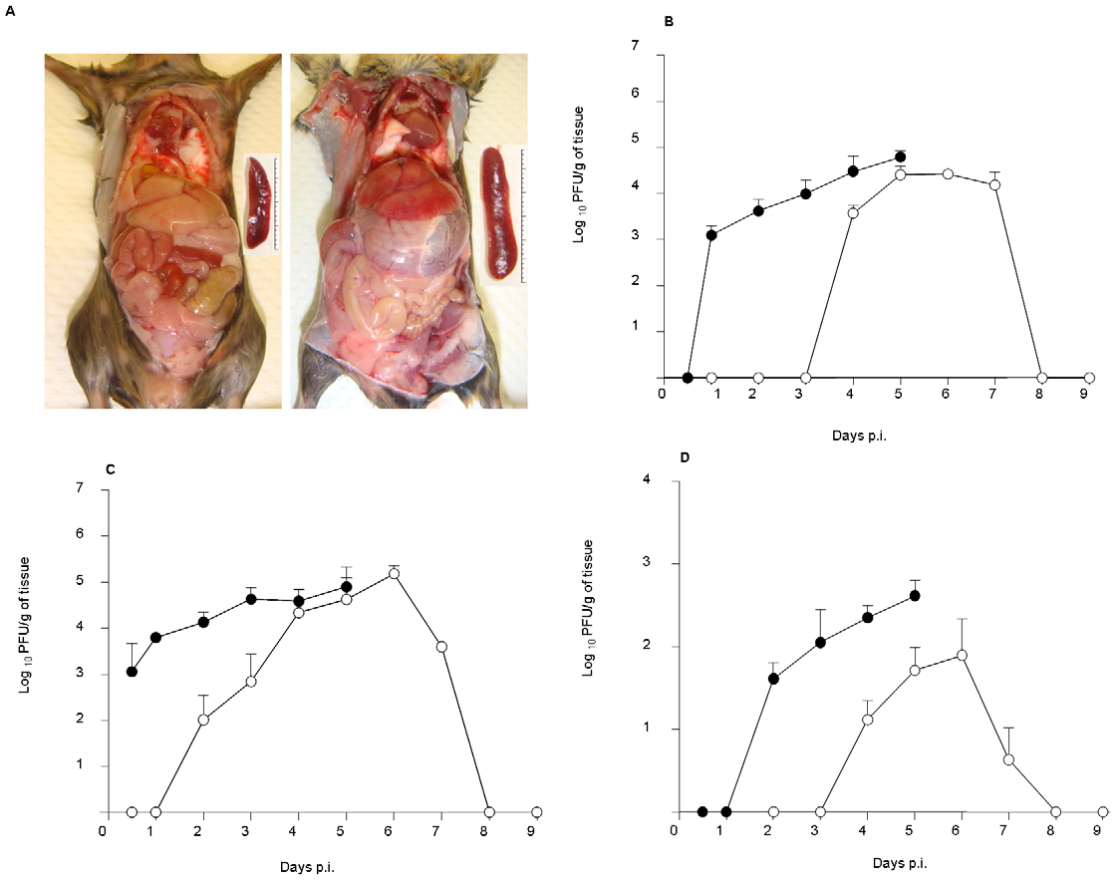
Pathology and virus titres in the liver, spleen and brain of D2Y98P-infected mice. (A) Mice were ip. infected with 10^7^ PFU of D2Y98P, and were sacrificed at moribund state and perfused extensively with PBS. Representative gross appearance of organs in the intraperitoneal cavity of uninfected (left panel) and ip. infected (right panel) mice. Insets highlight the difference in the spleen size between both animal groups. Virus titres were determined in the liver (B), spleen (C) and brain (D) from AG129 mice ip. infected with 10^7^ (black circle) or 10^4^ (open circle) PFU of D2Y98P virus. Results are expressed as log_10_ [mean ± SD] in PFU per gram of tissue. Five mice per time point per group were assessed. Results are representative of 2 independent experiments.

Gross pathological examination of the organs within the intraperitoneal cavity from moribund animals infected with 10^7^ PFU of D2Y98P revealed overt abnormalities that included a severely distended stomach, a significantly enlarged spleen and focal areas of haemorrhage in the liver, observable after systemic perfusion of the mice with saline (Fig. 4A). These features were not observed in moribund animals infected with 10^4^ PFU (data not shown).

Tissue tropism and kinetic of viral replication were determined in the intestines, liver, spleen, and brain from animals infected with either 10^7^ or 10^4^ PFU of D2Y98P. No infectious viral particles were detected in the intestines. In the spleen, liver and brain, the kinetic of the virus titers corresponded to the viremia profile; in animals infected with 10^7^ PFU, virus titres in the infected organs increased logarithmically in conjunction with disease advancement, reaching their highest at the time of death (Fig. 4B-D). Instead, in animals infected with 10^4^ PFU, the virus titres peaked at 5 or 6 days p.i. in the liver, spleen and brain, and progressively dropped until complete clearance by day 8 p.i. (Fig. 4B-D). Interestingly, the peak of virus titres achieved in the liver and spleen was comparable in both animal groups whereas peak titres in the brain (Fig. 4D) and plasma (Fig. 3A&B) were about 1 log higher in mice infected with 10^7^ PFU.

### Histological examination of organs from D2Y98P-infected mice

**Figure 5:**
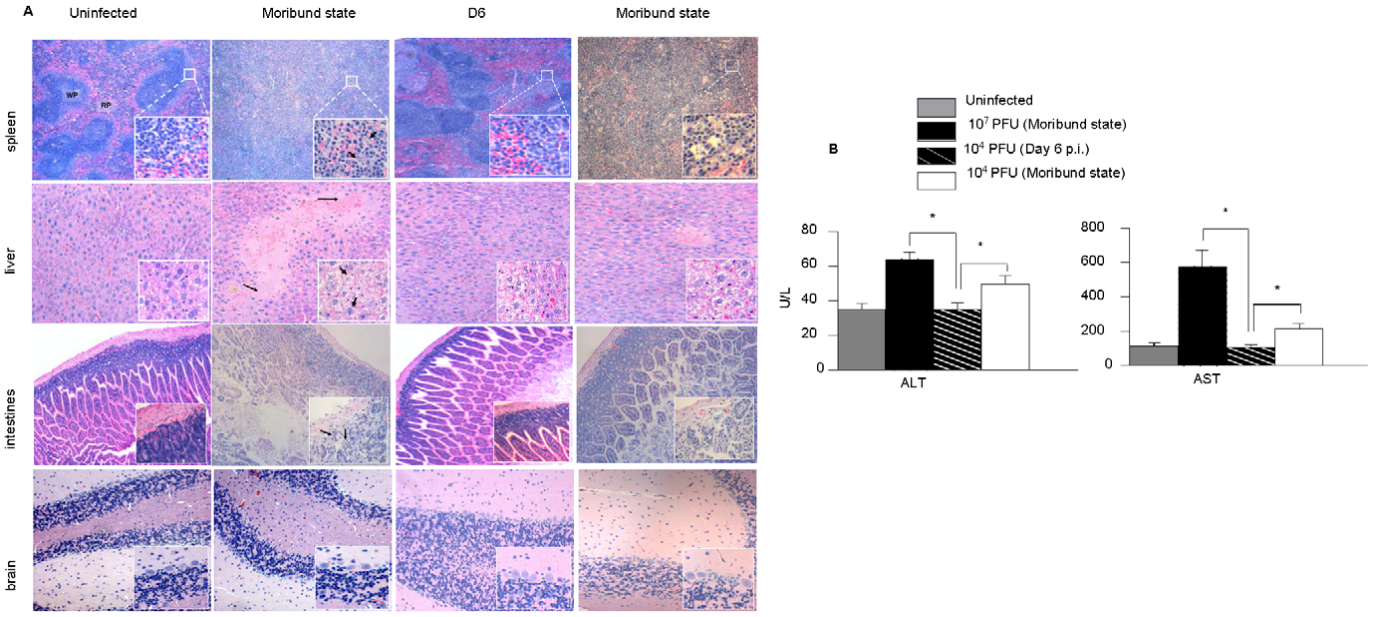
Histopathology of D2Y98P-infected mice. (A) Representative H&E-stained tissue sections from the spleen, liver, intestines and brain of AG129 mice ip. infected with 10^7^ or 10^4^ PFU of D2Y98P virus. Animals were euthanized at day 6 p.i. (10^4^ PFU dose) or at moribund state (10^4^ and 10^7^ PFU doses). Sections were viewed under a light microscope at 50x (spleen) or 100x (liver, intestines, brain) magnifications. Insets at the right bottom corners are observations made at 400x magnification. Representative sections from uninfected animals are shown in the left panels. Arrows indicate apoptotic debris (inset spleen), hemorrhage and edema (liver) or vacuolation of hepatocytes (inset liver), and inflammatory cells (inset intestines). Legend: RP, red pulp; WP, white pulp. (B) Serum levels of aspartate (AST) and alanine (ALT) transaminases. Mice were ip. infected with 10^7^ or 10^4^ PFU of D2Y98P. The animals were bled and euthanized at day 6 p.i. (10^4^ PFU) or at moribund state (10^4^ and 10^7^ PFU). Five mice per group and per time point were used. Results are expressed in U/L as the [mean ± SD] and are representative of 2 independent experiments.

Brain, spleen, liver and intestines were harvested from mice infected with 10^7^ or 10^4^ PFU of D2Y98P over the course of infection. Histological examination of H&E stained-sections from animals infected with 10^7^ PFU revealed progressive damage at both tissue and cellular levels which culminated at the time of death (Fig. 5A). The well defined limits of the splenic red and white pulp began to blur by day 3 p.i. (data not shown) and the spleen architecture was completely lost by day 5 p.i. (Fig. 5A). A larger magnification revealed the presence of apoptotic debris. The liver displayed focal areas of haemorrhage and edema of cell masses. Lymphoid aggregates and inflammatory infiltrates were also detected at the portal tract and within the sinusoidal spaces of the liver (data not shown). At the cellular level, extensive cytopathic effects that included hepatocyte swelling, cytoplasmic vacuolation and degeneration were observed. Liver damage was reflected by the significantly increased levels of aspartate (ALT) and alanine (AST) transaminases measured in the serum of the infected animals (Fig. 5B). Interestingly, despite the absence of detectable virus particles in the intestines, these tissues displayed marked infiltration of inflammatory cells and extensive architectural distortion at moribund state (Fig. 5A). Severe detachment and disintegration of the intestinal villi resulting in a debris-filled intestinal lumen was noted.

In animals infected with 10^4^ PFU of D2Y98P, no visible organ damage was noticeable at the peak of viremia, 6 days p.i. (Fig. 5A). However, at moribund state, the splenic architecture was severely impaired to an extent comparable to that observed in animals infected with 10^7^ PFU. In contrast, the liver and intestines were moderately affected with only localized areas of visible damage. Moderate but significant increase in the systemic levels of ALT and AST was measured at moribund state (Fig. 5B), indicative of some liver dysfunction. Apart from slight vascular congestion, brain sections from both animal groups did not display any significant pathological changes at any time post-infection (Fig. 5A).

### Vascular leakage in D2Y98P-infected mice

**Figure 6:**
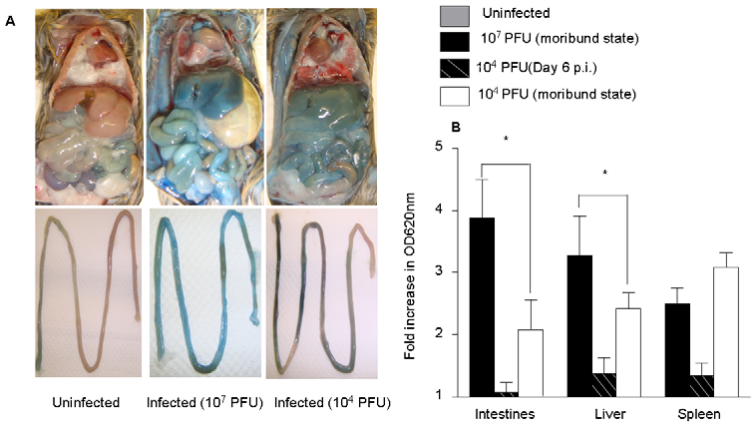
Vascular leakage in D2Y98P-infected mice. AG129 mice were inoculated ip. with 10^7^ or 10^4^ PFU of D2Y98P. At day 6 p.i. (10^4^ PFU dose) or at moribund state (both doses), mice were intravenously administered with Evans blue. After 2 hours, they were perfused extensively with PBS and assessed for Evans Blue extravasation in tissues. (A) Evan's blue extravasation in the peritoneal cavity (top panel) and intestines (bottom panel) of uninfected or D2Y98P-infected mouse at moribund state. (B) Quantification of Evans blue dye in the intestine, liver and spleen from mice. Five animals per group per time point were individually processed. Data are expressed as the [mean ± SD] of fold increases in OD_620nm_ per gram of wet tissue compared to uninfected controls. (C) Serum albumin concentration. Results are expressed as the [mean ± SD] of 5 animals per time point per group. *p<0.05. Results are representative of 2 independent experiments.

Vascular leakage, a hallmark of severe DEN infection in humans, was investigated in D2Y98P-infected AG129 mice using Evans blue dye extrusion assay [30], [37]. At moribund state, severe vascular leakage was observed (Fig. 6A) and measured (Fig. 6B) in the spleen, liver and intestines from animals infected with 10^7^ PFU compared to uninfected controls. Consistently, significant decreased levels in serum albumin were measured in these infected animals, indicative of plasmatic proteins leakage (Fig. 6C).

In animals infected with 10^4^ PFU, marginal dye extrusion was observed in the liver, intestines and spleen at the peak of viremia (6 days p.i.) whereas at moribund state, dye extrusion was markedly increased in all the organs examined (Fig. 6A&B). The extent of leakage in the liver and intestines was lesser than that observed in mice infected with 10^7^ PFU, whereas dye extrusion in the spleen was as high as in the animals infected with 10^7^ PFU (Fig. 6B). Interestingly, and in contrast to animals infected with 10^7^ PFU, serum albumin concentration measured in animals infected with 10^4^ PFU was significantly higher than that measured in uninfected control animals (Fig. 6C), suggestive of hemoconcentration.

### Cytokine expression levels in D2Y98P-infected mice

**Figure 7:**
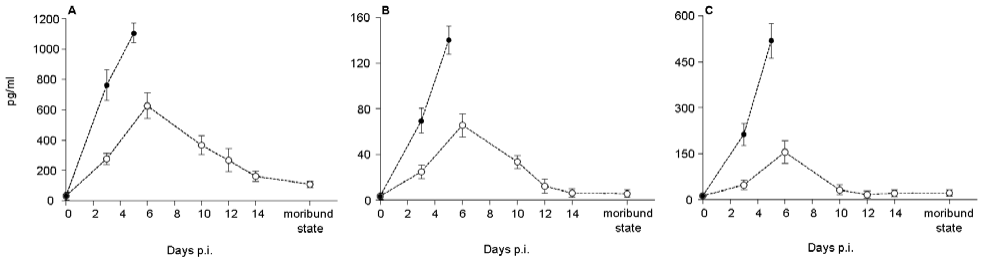
Pro-inflammatory cytokine expression in D2Y98P-infected mice. AG129 mice were ip. infected with 10^7^ or 10^4^ PFU of D2Y98P, bled at the indicated time points and immediately euthanized. Serum levels of IFN-γ (A), IL-6 (B) and TNF-α (C) were quantified. Results are expressed in pg/ml as the [mean ± SD] of 5 mice per time point and per group.

Enhanced cytokine production may lead to increased vascular permeability and has been proposed to contribute to DHF/DSS pathogenesis [39], [40]. The expression profile of three key pro-inflammatory cytokines, namely IFN-γ, IL-6 and TNF-α, was monitored over the course of infection in the serum of animals infected with 10^7^ or 10^4^ PFU of D2Y98P. In animals infected with 10^7^ PFU, the cytokine expression levels increased consistently over time and peaked at the time of death of the animals (Fig. 7). In contrast, in animals infected with 10^4^ PFU, the production of these pro-inflammatory cytokines corresponded to the viremia profile, peaking at day 6 p.i., followed by a progressive decline to reach basal production levels at moribund stage (Fig. 7). Of note, peak values of the systemic levels of these three cytokines were significantly higher in animals infected with 10^7^ PFU compared to animals infected with 10^4^ PFU.

### Hematology in D2Y98P-infected mice

Hematological disorders have been associated with DEN disease and tentatively used as diagnostic and prognostic markers [41], [42]. Total counts of red blood cells (RBC), white blood cells (WBC), lymphocytes, platelets and neutrophils were monitored in D2Y98P-infected mice over the course of infection (Table 1).

**Table 1:** Hematology in D2Y98P-infected mice.

Cell types	Uninfected	10^7^ PFU		10^4^ PFU		
		Day 3 p.i.	Moribund	Day 6 p.i.	Day 10 p.i.	Moribund
**WBC**	4.85 (0.54)	5.68 (0.68)	24.32* (1.61)	9.49* (1.23)	20.76* (4.75)	4.33 (0.73)
**NEU**	0.54 (0.09)	3.49* (0.21)	8.17* (1.14)	6.35* (1.19)	8.03* (2.67)	1.07* (0.09)
**LYM**	3.86 (0.36)	1.25* (0.11)	8.2* (2.07)	2.23* (0.31)	10.88* (2.34)	2.99 (1.00)
**RBC**	7.56 (0.31)	9.58* (0.27)	7.31 (0.53)	9.48* (0.54)	9.95* (0.21)	10.87* (0.43)
**HCT**	41.10 (1.2)	47.22* (1.14)	38.22 (2.6)	45.34* (2.36)	47.78* (0.92)	53.88* (1.11)
**PLT**	464.6 (65.4)	427.2 (26.1)	552.8 (43.3)	476.2 (23.33)	706.2* (40.85)	499.8 (42.87)

AG129 mice were ip. infected with 10^7^ or 10^4^ PFU of D2Y98P. At the indicated time points, 5 mice per group per time point were bled and euthanized. Blood samples were processed to determine white blood cells (WBC), neutrophils (NEU), lymphocytes (LYM), red blood cells (RBC), hematocrit (HCT), and platelets (PLT) counts. A group of uninfected mice was included as control. WBC, NEU, LYM and PLT counts are given in K/uL (10^3^ cells/ul), RBC count in M/uL (10^6^ cells/ul), and HCT in percentage (%). Data are expressed as the [mean ± SD] of individual measurements and are representative of 2 independent experiments. * p<0.05 compared to uninfected controls.

In animals infected with 10^7^ PFU, significant increase in RBC concentration and hematocrit was measured at day 3 p.i. compared to uninfected controls, indicative of hemoconcentration. At moribund state however (day 5 p.i.), the levels of RBC and hematocrit dropped, suggestive of hemorrhage. However, the levels of WBC, neutrophils and platelets increased substantially over time. Transient depletion in lymphocyte counts was observed at day 3 p.i. followed by significant increase at day 5 p.i.

In animals infected with 10^4^ PFU, progressive increase in RBC counts and hematocrit was observed over the course of infection, indicative of hemoconcentration. WBC, neutrophils, and platelets levels similarly increased progressively and reached peak values at 10 days p.i. At moribund state however, the levels measured were comparable to those measured in uninfected controls. Transient lymphopenia was observed at the peak of viremia (day 6 p.i.) followed by a very significant increase at day 10 p.i. Basal lymphocytes level was measured at moribund state.

Altogether, the hematological parameters indicate that infection with 10^7^ PFU of D2Y98P led to haemorrhage tendency, whereas infection with 10^4^ PFU resulted in hemoconcentration. Remarkably, no evidence of thrombocytopenia was observed in the infected animals as reflected by the platelets counts which were not found statistically different from the uninfected controls.

## Discussion

A growing number of immunocompetent, immunosuppressed and humanized mouse models of DEN infection have been explored, using an increasing number of mouse-adapted or cell-culture passaged DEN virus strains. However, none of these have so far managed to recapitulate all the clinical symptoms and manifestations of DEN disease as observed in humans. As humans and mosquitoes represent the only two natural hosts for DEN virus, it is unrealistic to hope address all the features of DEN pathogenesis in a single mouse model. However, previous studies have shown that it is possible to reproduce, and thus study, one or few aspects of DEN pathogenesis in a specific mouse model of DEN infection defined by a particular mouse background infected with a specific DEN virus strain through a particular route of administration and at a particular infectious dose. For example, a mouse model of DEN hemorrhage has recently been reported through intradermal infection of immunocompetent mice with a high dose of the non-mouse adapted DEN2 virus strain 16681 originally isolated from a DHF patient [43], [44]. Likewise, a humanized mouse strain infected subcutaneously with various DEN virus strains reportedly displayed clinical signs of DEN fever, including fever, viremia, erythema, and thrombocytopenia [45]. Similarly, the AG129 mouse model has allowed the investigation of some aspects of DEN pathogenesis including virus tropism, vascular leakage, and pathogenesis in context of a functional adaptive immune system [33]. Furthermore, the AG129 mouse background has proven useful for vaccine and drug testing [31], [32]. However, the lack of IFN α/β− and γ−signalling draws some limitations and calls for cautious interpretation of the findings and observations made in this mouse model. Furthermore, the susceptibility of AG129 mice to DEN infection appears to greatly depend on the DEN virus strain [32] and a limited number only have so far been reported to result in a productive infection with no, few or irrelevant clinical manifestations [30], [32]. Moreover, administration of high viral doses was necessary to trigger a virulent phenotype which resulted in animal death within few days at the peak of viremia [30].

Here we describe a non mouse-adapted DEN virus strain, D2Y98P, which is highly infectious in AG129 mice. D2Y98P is a serotype 2 DEN virus strain originally isolated in 1998 from a Singapore DEN-infected patient whose disease status at the time of sample collection, and disease outcome are unfortunately not known. The virus has been exclusively amplified in mosquito cells for less than 20 rounds. Interestingly, an earlier passage (P13) displayed a more attenuated virulent phenotype upon infection of AG129 mice (G. Tan, personal communication). This observation therefore suggests that mutation(s) have occurred in the viral genome upon amplification in mosquito cells that rendered the virus more virulent. Identification of the nucleotide changes between the two virus passages is currently in progress in our laboratory.

Infection of AG129 mice with a high dose (10^7^ PFU) of D2Y98P induced an acute lethal DEN infection where the peak of viremia and virus titres in the infected organs coincided with death of the animals, accompanied by cytokine storm, massive organ damage, and severe vascular damage leading to haemorrhage. It is thus likely that in this acute model of DEN infection, the pathological events are a consequence of both virus-induced cell death and massive inflammation reaction [39], [40]. Such virulent phenotype is similar to that described previously by Shresta and colleagues using the D2S10 DEN virus strain [30]. In contrast, infection of AG129 mice with a lower dose (10^4^ PFU) of D2Y98P led to a transient asymptomatic systemic viral infection followed by death of the animals few days after viral clearance, similar to the disease kinetic described in humans [3], [38]. A strong neutralizing IgG antibody response was measured in the infected animals and is likely to be involved in the viral clearance. Although increased vascular permeability (as indicated by increased serum albumin concentration and Evan's blue dye extrusion) was observed in the moribund animals, the actual cause of the animals' death remains elusive. Apparent destruction of the splenic architecture and liver dysfunction at moribund stage are likely to contribute to the sickness. Furthermore, as the disease progressed, infected animals appeared lethargic and displayed reduced motility. This may result in reduced water intake and dehydration of the animal, hence contributing to the sharp body weight loss observed towards moribund stage and consequently leading to animal death.

Widespread immune activation in response to acute DEN infection has been well documented in DEN patients, and circulating levels of various pro-inflammatory cytokines were found to be elevated in patients with severe DEN [40]. Likewise, the levels of three key pro-inflammatory cytokines implicated in DF/DHF, namely IL-6, TNF-α and IFN-γ, were significantly elevated in the D2Y98P-infected AG129 mice and were directly dependent on the initial infectious dose. Consistently, extensive damage of various organs including the spleen, liver and intestines was observed in animals infected with a high viral dose (10^7^ PFU). In contrast, lower levels of cytokine production in animals infected with a low viral dose (10^4^ PFU) correlated with milder organ damage except for the spleen that appeared at moribund stage, to be as extensively damaged as in animals infected with a high viral dose; the absence of infectious viral particles in the moribund animals excludes a direct virus cytopathic effect but rather suggests some immunological disorder that may arise from the overstimulation of immune cells possibly by persistent viral antigens.

In contrast to the liver and spleen, no histological damage or abnormalities were detected in the brain of animals infected with 10^7^ PFU or 10^4^ PFU, although infectious viral particles were readily detected in this tissue after systemic perfusion. This observation suggests that the virus is capable of extravasating from the systemic circulation and cross the blood-brain barrier but may not effectively replicate in the brain. Therefore, in this mouse model, and as reported in dengue patients [46], [47], meningitis and/or encephalitis may not contribute significantly to disease severity.

The action of a variety of cytokines, chemokines, and other soluble mediators on endothelial cells has been proposed to affect vascular permeability during DEN infection [39]. Vascular leakage is a hallmark of DHF/DSS leading to hemoconcentration and hemorrhagic manifestations [41], [48], as observed in mice infected with 10^7^ PFU of D2Y98P for whom focal areas of haemorrhage were observed in the liver, and low hematocrit and serum albumin levels were measured. In this animal group, high levels of pro-inflammatory cytokines are likely responsible for the observed severe vascular leakage, particularly in the intestines where no infectious viral particles were detected.

However, in mice infected with 10^4^ PFU, neither significant vascular leakage nor hemorrhage was detected at the peak of viremia despite elevated levels of IFN-γ, IL-6 and TNF-α. Instead, increased vascular permeability was clearly observed at moribund stage where the production of these three cytokines has returned to basal level. This observation suggests that other pro-inflammatory cytokines may be involved in the increased vascular permeability observed in this low viral dose infection model. Indeed, in addition to IFN-γ, IL-6 and TNF-α, a number of cytokines, chemokines and other soluble mediators have been demonstrated or proposed to play a role in vascular leakage in DEN disease [39]. Alternatively or additionally, other mediators previously proposed to increase vascular permeability such as immune complexes [49], nitrite oxide production [39], or cross-reactive anti-NS1 antibodies [6], [7], may be at play. Furthermore, hemoconcentration and increased serum albumin level suggests that fluid only but not proteins or cells, leaks from the blood vessels. Increased vascular permeability without morphological damage of the capillary endothelium is believed to be the cardinal feature of DSS [39], [49] and thus appears to be reproduced in this mouse model of DEN infection. Further investigation is however needed to decipher the actual mechanisms underlying this phenomenon.

Remarkably, thrombocytopenia, a hallmark of severe disease in DEN patients, was not detected in the animals infected with D2Y98P virus, regardless of the initial infectious dose. Transient drop in platelet counts has been previously observed in a number of mouse models of DEN infection [15] including AG129 [33], ruling out the possibility that the lack of IFNγ signalling in these mice would impair the mechanism(s) involved in thrombocytopenia. The absence of thrombocytopenia in our model may thus be inherent to the D2Y98P virus strain. A number of immunological mechanisms and effectors have been proposed to play a role in thrombocytopenia during DEN infection [50]–[53], but the differential ability of DEN virus strains to induce thrombocytopenia in a single model of DEN infection has never been investigated.

In conclusion, the attractiveness of the D2Y98P strain lies in its ability to induce, without the need for mouse-adaptation and upon peripheral administration of a low viral dose, a virulent phenotype in AG129 mice with a productive viral replication and dissemination accompanied by some relevant clinical manifestations, including disease kinetic, organ damage/dysfunction and increased vascular permeability. This model thus offers the opportunity to further dissect some of the mechanisms involved in DEN pathogenesis with the caveat that AG129 mice are defective in IFN signalling. Furthermore, the induction of a disease kinetic where the time-of-death window is distinct from the viremic phase makes this low viral dose model unique and an attractive platform for assessing the efficacy of DEN vaccine and drug candidates.
