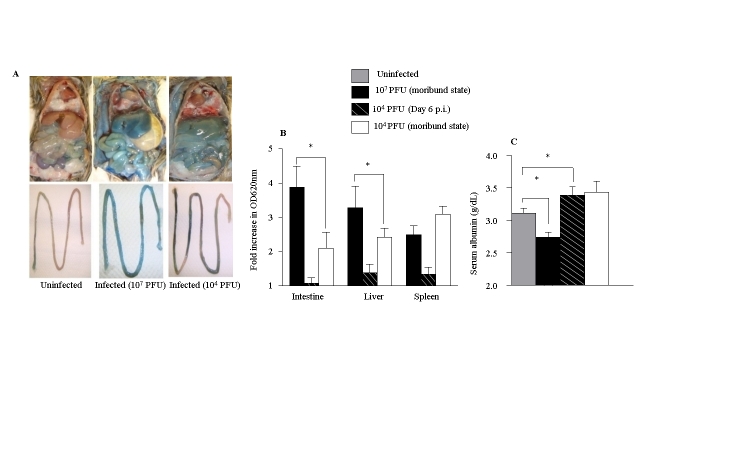# Correction: A Non Mouse-Adapted Dengue Virus Strain as a New Model of Severe Dengue Infection in AG129 Mice

**DOI:** 10.1371/annotation/03774b36-c453-404a-b295-7b91bfd9cebd

**Published:** 2010-10-27

**Authors:** Grace K. Tan, Jowin K. W. Ng, Scott L. Trasti, Wouter Schul, George Yip, Sylvie Alonso

Part C in Figure 6 was missing. See the correct figure here: 

**Figure:**